# Incidental Asymptomatic Giant Hydatid Cyst of the Interventricular Septum Bulging Into the Right Ventricle

**DOI:** 10.7759/cureus.13532

**Published:** 2021-02-24

**Authors:** Ramia Bougrine, Hanane Aissaoui, Noha Elouafi, Nabila Ismaili

**Affiliations:** 1 Cardiology, Mohammed I University/Mohammed VI University Hospital, Oujda, MAR; 2 Cardiology, Mohammed I University/Mohammed VI University Hospital/Epidemiological Laboratory of Clinical Research and Public Health, Oujda, MAR

**Keywords:** hydatid cyst, echinococcus granulosus, asymptomatic, ventricular septum

## Abstract

Hydatid disease is caused by the larvae of Echinococcus granulosus. Domestic animals like cats and dogs are the primary carriers of echinococcal organisms. This parasitosis is still endemic in some particular regions of the world. The cardiac hydatid cyst is an exceptional infection. We report a case of an asymptomatic giant cardiac hydatid cyst in the interventricular septum (IVS) protruding in the right ventricular diagnosed incidentally by scan tomography during acute pancreatitis emergency. Transthoracic echocardiography revealed a cystic mass in the IVS bulging into the right ventricle. The diagnosis was confirmed by a cardiac CT scan.

## Introduction

Hydatidosis is a zoonotic infection caused mainly by accidental ingestion of Echinococcus granulosus eggs from the dog (the definitive host). The human remains an intermediate host [1]. Contamination occurs through direct contact with the dog or indirect through ingestion of food contaminated by parasitic eggs [2,3]. The organs most affected are the liver (50%-70% of cases) and lungs (20%-30%) [4]. Smooth muscles, the skeleton and hydatid heart disease are extremely rare locations in less than 2% of cases [3,5]. Cardiac involvement represents only 0.5% to 2% of patients with hydatid disease [6,7].

Hydatid cyst of the interventricular septum (IVS) is one of the most infrequent locations of cardiac involvement at 4% of cardiac location, the clinical presentation ranges from asymptomatic to sudden death. Computed tomography and MRI are essential for definitive diagnosis and planning for surgery [2].

We report a giant asymptomatic cardiac hydatid cyst involving the IVS and protruding into the right ventricle.

## Case presentation

**Figure 1:**
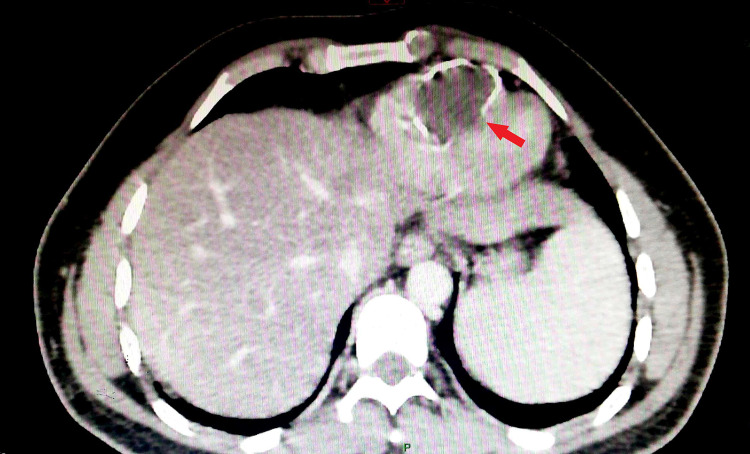
Abdominal tomography imaging showing the huge interventricular cyst bulging into the right ventricle.

**Figure 2:**
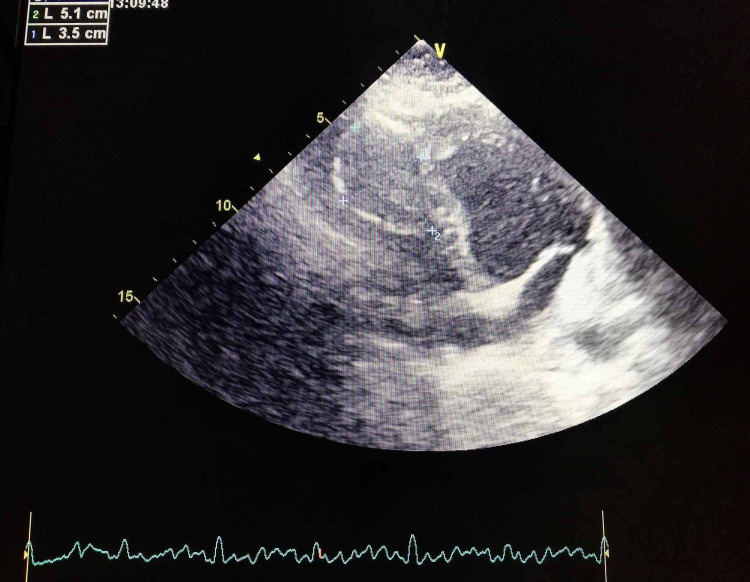
Transthoracic echocardiography showing the hydatid cyst in the interventricular septum.

**Figure 3:**
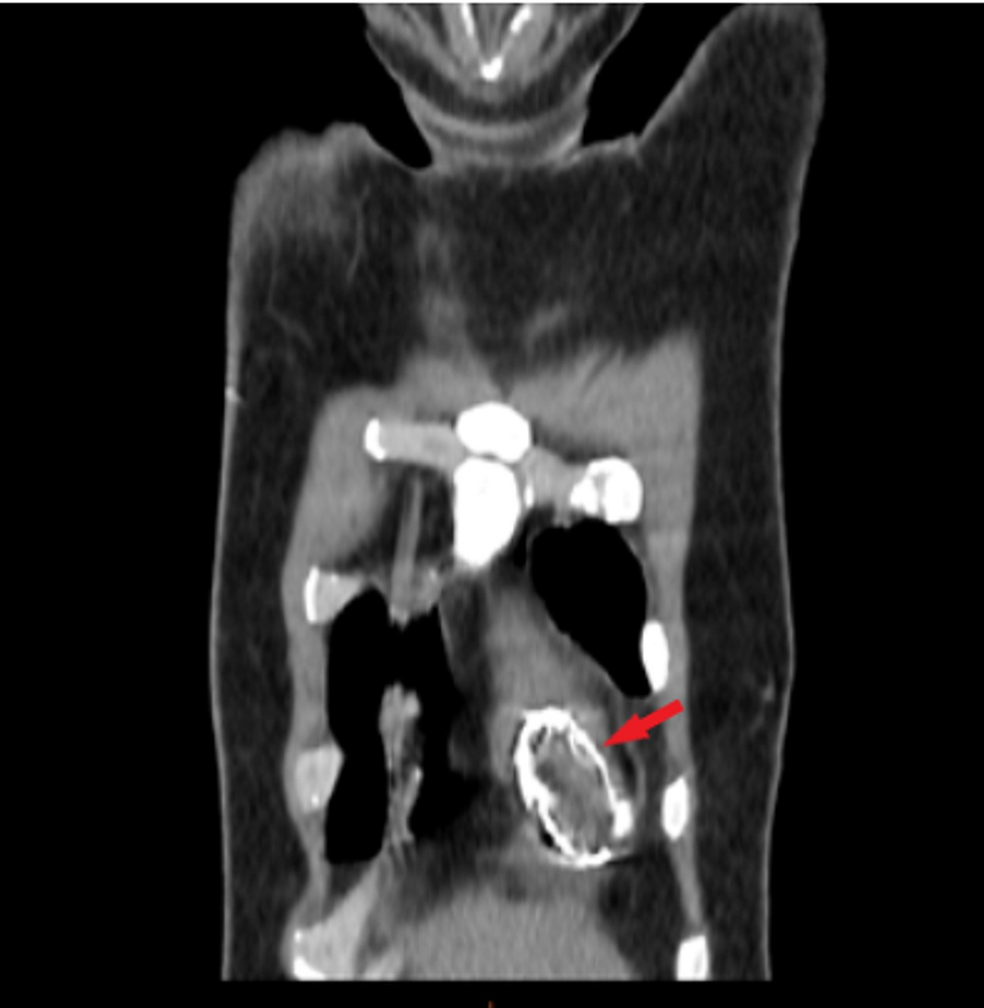
Thoracic tomography showing the intracardiac hydatic cyst.

A 36-year-old man who lives in a rural area of the north of Africa was admitted to the emergency department for intense epigastric pain, with a past medical history of liver hydatid cyst (at the age of seven years) and total pericystectomy of a hydatid cyst of tibialis posterior muscle a few months ago. Physical examination showed an abdominal tenderness with normal cardiovascular auscultation. Laboratory analysis revealed a high level of lipasemia (880 UI/L), a normal level of CRP (2.3 mg/l < 6 mg/l normal range), hypereosinophilia (700 elements/mm^3^). Computed tomographic (CT) images with contrast injection revealed acute pancreatitis (stage C) and incidental cardiac intra-ventricular cyst (Figure 1). Transthoracic echocardiography revealed a normal ejection fraction (EF: 65%) with a huge cyst masse 51x35 mm (Figure 2) in the IVS protruding into the right ventricle without outflow obstruction and a normal right function. We completed with trans esophageal echocardiography revealing a calcified hydatid cyst settled in the middle of the IVS. Thoracic CT confirmed a calcified cardiac hydatid cyst measuring 36/40/48 mm (Figure 3). A serum anti-echinococcus antibody test was positive, and no other cyst location was detected. Currently in a body scan, and an electrocardiogram Holter ECG monitor revealed sinus tachycardia without arrhythmia, the patient refuses surgical treatment, we are suitable for strict surveillance with a favorable outcome after 16 months.

## Discussion

Left ventricle is the most common area affected by the hydatic infection disease (55%-60%), followed by the IVS and the right atrium [8]. The low incidence of this localization is explained by rhythmic contractions of the heart which provide natural resistance to the presence of a viable hydatid cyst. However, when this mechanism fails, the pathways of cardiac invasion by the parasite are through the left side of the heart with coronary or pulmonary circulation or by direct extension from adjacent structures [9].

The natural evolution of hydatic cyst will eventually invade the surrounding structures, can obstruct blood flow, when also invade the conductive system of the heart can cause atrioventricular block [1], even can mimic acute coronary syndrome by compressing the coronary arteries [3]. The clinical presentation of cardiac hydatid cyst is highly variable, ranging from being asymptomatic to sudden death [5].

Diagnosis can be difficult. Echocardiography is a non-invasive method, very sensitive, and easily performed in the first-line examination method. The echocardiographic appearance of a hydatid cyst may be confused with heart tumors. In this case, it is necessary to supplement with computed tomography or MRI which are effective in establishing a differential diagnosis between tumors and hydatid cysts [10]. In our case, the diagnosis was easy given the rich history of hydatid disease.

The most important complication is the rupture of the cyst [11] and may trigger anaphylactic shock or tamponade, systemic or pulmonary embolization, and compression of coronary branches [6].

Surgery is the therapy of choice for cardiac hydatid disease to allow a fast recovery and avoid postoperative complications even in asymptomatic patients [7]; however, adhesion to vital structures sometimes makes this impossible with a lot of complications, as in our case. Albendazole therapy is typically prescribed for at least four days preoperatively and for 4 to 12 weeks postoperatively [6].

Thus, in view of the calcified character of the cyst and an asymptomatic patient who still refuses surgery we decided to clinical follow-up.

Our patient was asymptomatic during follow-up, and echocardiography after 16 months showed a stable right ventricular mass. Non-surgical management may be beneficial in a selected group of patients.

Hygiene education and healthy practices with animals especially pets can reduce the prevalence of the disease.

## Conclusions

In conclusion, cardiac hydatid cyst is a critical variant of parasitic infection, it presents in various ways. Early diagnosis and surgical intervention are essential even in asymptomatic patients to prevent major complications. Non-surgical management can be beneficial in a high-risk population. Our case shows the benefit of close-up supervision in the therapeutic strategy and long-term prognosis.

## References

[1] Joseph AG, Lahiri R, Sengupta G (2020). Giant hydatid cyst of interventricular septum of heart. Indian J Thoracic Cardiovasc Surg.

[2] Shojaei E, Yassin Z, Rezahosseini O (2016). Cardiac hydatid cyst: a case report. Iran J Public Health.

[3] Besir Y, Gucu A, Surer S, Rodoplu O, Melek M, Tetik O (2013). Giant cardiac hydatid cyst in the interventricular septum protruding to right ventricular epicardium. Indian Heart J.

[4] Tekin AF, Durmaz MS,  Dağli M (2018). Left ventricular hydatid cyst mimicking acute coronary syndrome. Radiology Case Rep.

[5] Salih Abdulwahid M, Kakamad Fahmi H, Salih Rawezh Q (2018). Hydatid cyst of the thigh: a case report with literature review. Int J Surg Case Rep.

[6] Ipek G, Omeroglu SN, Goksedef D (2011). Large cardiac hydatid cyst in the interventricular septum. Tex Heart Inst J.

[7] Tetik O, Yılık L, Emrecan B, Ozbek C, Gürbüz A (2002). Giant hydatid cyst in the interventricular septum of a pregnant woman. Tex Heart Inst J.

[8] Ohri S, Sachdeva A, Bhatia M, Shrivastava S (2015). Cardiac hydatid cyst in left ventricular free wall. Echo Res Pract.

[9] Shehatha J, Alward M, Saxena P, Konstantinov IE (2009). Surgical management of cardiac hydatidosis. Tex Heart Inst J.

[10] Kankilic N, Aydin MS, Günendi T, Göz M (2020). Unusual hydatid cysts: cardiac and pelvic-ilio-femoral hydatid cyst case reports and literature review. Braz J Cardiovasc Surg.

[11] Ilic S, Parezanovic V, Djukic M, Kalangose A (2008). Ruptured hydatid cyst of the interventricular septum with acute embolic pulmonary artery complications. Pediatr Cardiol.

